# Hexyl (*E*)-3-(3,4-dihy­droxy­phen­yl)acrylate

**DOI:** 10.1107/S1600536811051671

**Published:** 2011-12-10

**Authors:** Jun Wang, Shuang-Shuang Gu, Jing Li, Fu-An Wu

**Affiliations:** a School of Biological and Chemical Engineering, Jiangsu University of Science and Technology, Zhenjiang 212018, People’s Republic of China; b Sericultural Research Institute, Chinese Academy of Agricultural Sciences, Zhenjiang 212018, People’s Republic of China

## Abstract

The title mol­ecule, C_15_H_20_O_4_, has an *E* conformation about its C=C bond and is almost planar (r.m.s. deviation of all non-H atoms = 0.04 Å). The crystal structurere features O—H⋯O and C—H⋯O hydrogen bonds.

## Related literature

For general background to caffeic acid and its derivatives, see: Buzzi *et al.* (2009 ▶); Uwai *et al.* (2008 ▶). For details of the synthesis, see: Feng *et al.* (2011 ▶); Son *et al.* (2011 ▶). For related structures, see: Xia *et al.* (2004 ▶, 2006 ▶). For standard bond lengths, see: Allen *et al.* (1987 ▶).
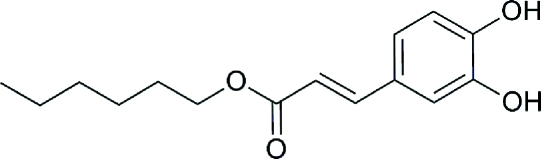


## Experimental

### 

#### Crystal data

C_15_H_20_O_4_
*M* *_r_* = 264.31
Triclinic, 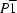

*a* = 5.2920 (11) Å
*b* = 10.689 (2) Å
*c* = 12.732 (3) Å
α = 95.45 (3)°
β = 92.76 (3)°
γ = 96.84 (3)°
*V* = 710.6 (2) Å^3^
*Z* = 2
Mo *K*α radiation
μ = 0.09 mm^−1^
*T* = 293 K
0.20 × 0.10 × 0.10 mm

#### Data collection

Enraf–Nonius CAD-4 diffractometer
Absorption correction: ψ scan (North *et al.*, 1968 ▶) *T*_min_ = 0.983, *T*_max_ = 0.991
2912 measured reflections
2608 independent reflections
1515 reflections with *I* > 2σ(*I*)
*R*_int_ = 0.022
3 standard reflections every 200 reflections  intensity decay: 1%

#### Refinement

*R*[*F* ^2^ > 2σ(*F* ^2^)] = 0.061
*wR*(*F* ^2^) = 0.178
*S* = 1.00
2608 reflections
172 parameters
H-atom parameters constrained
Δρ_max_ = 0.19 e Å^−3^
Δρ_min_ = −0.28 e Å^−3^

### 

Data collection: *CAD-4 EXPRESS* (Enraf–Nonius, 1994 ▶); cell refinement: *CAD-4 EXPRESS*; data reduction: *XCAD4* (Harms & Wocadlo, 1995 ▶); program(s) used to solve structure: *SHELXS97* (Sheldrick, 2008 ▶); program(s) used to refine structure: *SHELXL97* (Sheldrick, 2008 ▶); molecular graphics: *SHELXTL* (Sheldrick, 2008 ▶); software used to prepare material for publication: *PLATON* (Spek, 2009 ▶).

## Supplementary Material

Crystal structure: contains datablock(s) I, global. DOI: 10.1107/S1600536811051671/aa2034sup1.cif

Structure factors: contains datablock(s) I. DOI: 10.1107/S1600536811051671/aa2034Isup2.hkl

Supplementary material file. DOI: 10.1107/S1600536811051671/aa2034Isup3.cml

Additional supplementary materials:  crystallographic information; 3D view; checkCIF report

## Figures and Tables

**Table 1:** Hydrogen-bond geometry (Å, °)

*D*—H⋯*A*	*D*—H	H⋯*A*	*D*⋯*A*	*D*—H⋯*A*
O1—H1*A*⋯O2^i^	0.85	2.07	2.857 (2)	154
O2—H2*A*⋯O3^ii^	0.82	1.97	2.786 (3)	173
C5—H5*A*⋯O3^ii^	0.93	2.54	3.243 (3)	133

Symmetry codes: (i) 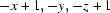; (ii) 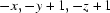.

**Fig. 1.**
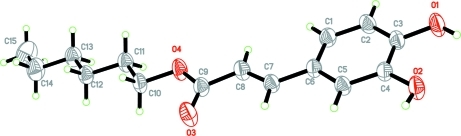
The molecular structure of the title molecule, with the atom numbering scheme. Thermal displacement ellipsoids are drawn at 30% probability level.

**Fig. 2.**
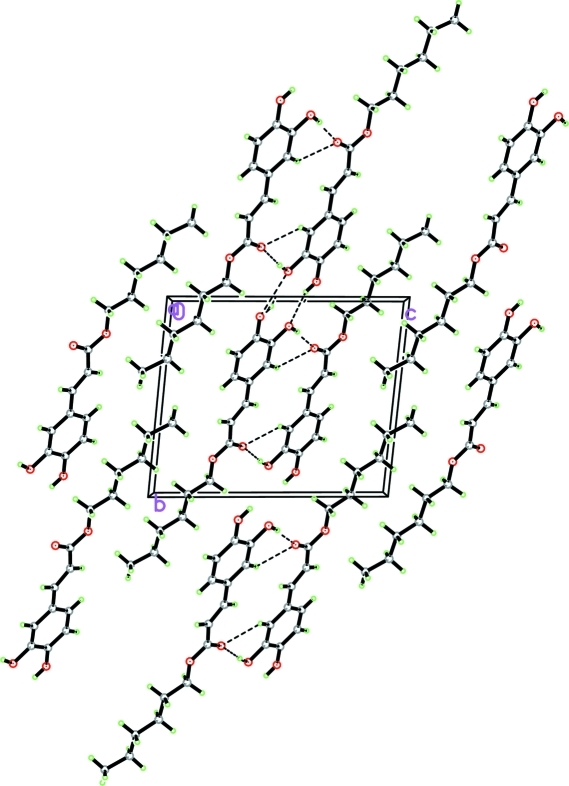
A partial packing diagram. Hydrogen bonds are shown as dashed lines.

## supplementary crystallographic information

### Comment

Caffeic acid and its derivatives are widely distributed in medicinal plants and are therefore present in human plasma in a diet dependent concentration. These compounds are known to show a variety of biological effects such as anti-tumor, anti-oxidant, and anti-inflammatory activities (Uwai *et al.*, 2008; Buzzi *et al.*, 2009). In order to investigate their properties better, we synthesize a series of caffeic acid esters. The title compound, hexyl (*E*)-3-(3,4-dihydroxyphenyl)acrylate (I) was obtained earlier (Feng *et al.*, 2011; Son *et al.*, 2011). We report herein the crystal structure of the title compound.

The molecule of (I) has an *E* configuration (Fig. 1). All non-H atoms of (I) are almost coplanar, with a root mean square deviation from the least-squares plane of 0.04 A°. The bond lengths and angles are within normal ranges (Allen *et al.*, 1987), they are in very good agreement with those found in similar caffeic acid structures (Xia *et al.*, 2004; Xia *et al.*, 2006)

In the crystal structure, intermolecular O—H···O interactions (Table 1) link the molecules into ribbons parallel to the (112) plane (Fig. 2), this may be effective in the stabilization of the structure. On the other hand, the intramolecular O—H···O H-bond also contribute to the stability of the molecular configuration (Table 1).

### Experimental

Esterification of caffeic acid with hexyl alcohol was performed in a column (inner diameter = 15 mm, length = 200 mm). Cation exchange resin CD-552 particles (5 g), molecular sieve (5 g) and glass beads of 2 mm in diameter were packed into the middle of the reactor. In a reaction mixture tank, caffeic acid (8.95 g) was mixed with 100 ml of hexyl alcohol. The reaction mixture was supplied to the reaction column at 10.0 ml/h. The reaction continued at 90°C for 24 h. The mixture was evaporated to dryness and followed by the addition of ethanol and extracted with dichloromethane three times. The dichloromethane extract was evaporated to give a solid residue. The residue was recrystallized from ethanol/petroleum ether (1:1) to give the title compound as brown crystals (4.9 g, 54.7%).

### Refinement

The H atoms were placed in calculated positions (O—H = 0.82 A ° and C—H = 0.93–0.97 A °) and constrained to ride on their parent atoms, with *U*_iso_(H) = 1.2 or 1.5*U*eq(C, O).

### Crystal data

**Table:**

C_15_H_20_O_4_	*Z* = 2
*M**_r_* = 264.31	*F*(000) = 284
Triclinic, *P*1	*D*_x_ = 1.235 Mg m^−^^3^
Hall symbol: -P 1	Mo *K*α radiation, λ = 0.71073 Å
*a* = 5.2920 (11) Å	Cell parameters from 25 reflections
*b* = 10.689 (2) Å	θ = 9–13°
*c* = 12.732 (3) Å	µ = 0.09 mm^−^^1^
α = 95.45 (3)°	*T* = 293 K
β = 92.76 (3)°	Block, brown
γ = 96.84 (3)°	0.20 × 0.10 × 0.10 mm
*V* = 710.6 (2) Å^3^	

### Data collection

**Table:**

Enraf–Nonius CAD-4 diffractometer	1515 reflections with *I* > 2σ(*I*)
Radiation source: fine-focus sealed tube	*R*_int_ = 0.022
graphite	θ_max_ = 25.4°, θ_min_ = 1.6°
ω/2θ scans	*h* = 0→6
Absorption correction: ψ scan (North *et al.*, 1968)	*k* = −12→12
*T*_min_ = 0.983, *T*_max_ = 0.991	*l* = −15→15
2912 measured reflections	3 standard reflections every 200 reflections
2608 independent reflections	intensity decay: 1%

### Refinement

**Table:**

Refinement on *F*^2^	Primary atom site location: structure-invariant direct methods
Least-squares matrix: full	Secondary atom site location: difference Fourier map
*R*[*F*^2^ > 2σ(*F*^2^)] = 0.061	Hydrogen site location: inferred from neighbouring sites
*wR*(*F*^2^) = 0.178	H-atom parameters constrained
*S* = 1.00	*w* = 1/[σ^2^(*F*_o_^2^) + (0.085*P*)^2^ + 0.096*P*] where *P* = (*F*_o_^2^ + 2*F*_c_^2^)/3
2608 reflections	(Δ/σ)_max_ < 0.001
172 parameters	Δρ_max_ = 0.19 e Å^−^^3^
0 restraints	Δρ_min_ = −0.28 e Å^−^^3^

### Special details

**Table:**

Geometry. All e.s.d.'s (except the e.s.d. in the dihedral angle between two l.s. planes) are estimated using the full covariance matrix. The cell e.s.d.'s are taken into account individually in the estimation of e.s.d.'s in distances, angles and torsion angles; correlations between e.s.d.'s in cell parameters are only used when they are defined by crystal symmetry. An approximate (isotropic) treatment of cell e.s.d.'s is used for estimating e.s.d.'s involving l.s. planes.
Refinement. Refinement of *F*^2^ against ALL reflections. The weighted *R*-factor *wR* and goodness of fit *S* are based on *F*^2^, conventional *R*-factors *R* are based on *F*, with *F* set to zero for negative *F*^2^. The threshold expression of *F*^2^ > σ(*F*^2^) is used only for calculating *R*-factors(gt) *etc*. and is not relevant to the choice of reflections for refinement. *R*-factors based on *F*^2^ are statistically about twice as large as those based on *F*, and *R*- factors based on ALL data will be even larger.

### Fractional atomic coordinates and isotropic or equivalent isotropic displacement parameters (Å^2^)

**Table:**

	*x*	*y*	*z*	*U*_iso_*/*U*_eq_	
O1	0.7392 (4)	0.09426 (17)	0.38828 (16)	0.0649 (6)	
H1A	0.6881	0.0408	0.4304	0.078*	
C1	0.5906 (5)	0.3938 (2)	0.3065 (2)	0.0504 (7)	
H1B	0.6496	0.4508	0.2597	0.060*	
O2	0.3463 (4)	0.13532 (16)	0.51361 (14)	0.0564 (6)	
H2A	0.2394	0.1617	0.5516	0.085*	
C2	0.7038 (5)	0.2853 (2)	0.3144 (2)	0.0528 (8)	
H2B	0.8390	0.2700	0.2730	0.063*	
C3	0.6192 (5)	0.1991 (2)	0.3832 (2)	0.0463 (7)	
O3	0.0120 (4)	0.75695 (18)	0.36537 (16)	0.0685 (7)	
O4	0.2768 (4)	0.81988 (16)	0.24479 (15)	0.0571 (6)	
C4	0.4190 (5)	0.2232 (2)	0.4456 (2)	0.0434 (7)	
C5	0.3053 (5)	0.3312 (2)	0.43703 (19)	0.0440 (7)	
H5A	0.1696	0.3462	0.4783	0.053*	
C6	0.3883 (5)	0.4188 (2)	0.36794 (19)	0.0420 (6)	
C7	0.2635 (5)	0.5329 (2)	0.36356 (19)	0.0443 (7)	
H7A	0.1264	0.5394	0.4059	0.053*	
C8	0.3218 (5)	0.6278 (2)	0.3067 (2)	0.0505 (7)	
H8A	0.4555	0.6237	0.2622	0.061*	
C9	0.1864 (5)	0.7388 (2)	0.3105 (2)	0.0466 (7)	
C10	0.1571 (6)	0.9344 (2)	0.2397 (2)	0.0518 (7)	
H10A	0.1845	0.9868	0.3067	0.062*	
H10B	−0.0249	0.9137	0.2235	0.062*	
C11	0.2783 (6)	1.0026 (2)	0.1537 (2)	0.0525 (7)	
H11A	0.2586	0.9468	0.0884	0.063*	
H11B	0.4594	1.0242	0.1720	0.063*	
C12	0.1614 (5)	1.1224 (2)	0.1367 (2)	0.0506 (7)	
H12A	−0.0210	1.1008	0.1222	0.061*	
H12B	0.1877	1.1792	0.2015	0.061*	
C13	0.2692 (6)	1.1911 (3)	0.0477 (2)	0.0584 (8)	
H13A	0.2456	1.1339	−0.0168	0.070*	
H13B	0.4512	1.2140	0.0629	0.070*	
C14	0.1498 (7)	1.3098 (3)	0.0292 (3)	0.0744 (10)	
H14A	−0.0338	1.2884	0.0198	0.089*	
H14B	0.1860	1.3702	0.0915	0.089*	
C15	0.2443 (8)	1.3718 (3)	−0.0660 (3)	0.0952 (13)	
H15A	0.1629	1.4464	−0.0732	0.143*	
H15B	0.2044	1.3136	−0.1284	0.143*	
H15C	0.4256	1.3947	−0.0569	0.143*	

### Atomic displacement parameters (Å^2^)

**Table:**

	*U*^11^	*U*^22^	*U*^33^	*U*^12^	*U*^13^	*U*^23^
O1	0.0696 (14)	0.0486 (12)	0.0879 (15)	0.0312 (10)	0.0301 (12)	0.0239 (10)
C1	0.0538 (17)	0.0444 (15)	0.0573 (17)	0.0095 (13)	0.0182 (14)	0.0156 (13)
O2	0.0697 (13)	0.0446 (11)	0.0651 (12)	0.0252 (10)	0.0277 (10)	0.0239 (9)
C2	0.0500 (17)	0.0478 (16)	0.0664 (19)	0.0191 (14)	0.0213 (15)	0.0108 (14)
C3	0.0475 (16)	0.0373 (14)	0.0569 (17)	0.0139 (12)	0.0091 (13)	0.0056 (12)
O3	0.0834 (16)	0.0585 (13)	0.0778 (14)	0.0343 (11)	0.0419 (12)	0.0294 (11)
O4	0.0708 (13)	0.0429 (11)	0.0678 (13)	0.0232 (10)	0.0280 (11)	0.0254 (10)
C4	0.0489 (16)	0.0362 (14)	0.0478 (15)	0.0100 (12)	0.0100 (13)	0.0085 (12)
C5	0.0484 (16)	0.0406 (15)	0.0470 (15)	0.0153 (12)	0.0148 (13)	0.0077 (12)
C6	0.0485 (16)	0.0359 (14)	0.0441 (14)	0.0104 (12)	0.0083 (12)	0.0084 (11)
C7	0.0486 (17)	0.0403 (15)	0.0477 (16)	0.0126 (13)	0.0137 (13)	0.0083 (12)
C8	0.0581 (18)	0.0464 (16)	0.0533 (17)	0.0174 (14)	0.0213 (14)	0.0150 (13)
C9	0.0539 (17)	0.0393 (15)	0.0509 (16)	0.0126 (13)	0.0110 (14)	0.0134 (12)
C10	0.0632 (19)	0.0366 (14)	0.0618 (18)	0.0199 (13)	0.0177 (15)	0.0136 (13)
C11	0.0647 (19)	0.0437 (15)	0.0538 (16)	0.0150 (14)	0.0165 (14)	0.0133 (13)
C12	0.0589 (18)	0.0412 (15)	0.0556 (17)	0.0119 (13)	0.0149 (14)	0.0124 (13)
C13	0.072 (2)	0.0495 (17)	0.0578 (18)	0.0131 (15)	0.0152 (16)	0.0156 (14)
C14	0.098 (3)	0.0557 (19)	0.077 (2)	0.0170 (18)	0.018 (2)	0.0281 (17)
C15	0.134 (4)	0.072 (2)	0.083 (3)	0.002 (2)	0.007 (2)	0.037 (2)

### Geometric parameters (Å, °)

**Table:**

O1—C3	1.357 (3)	C8—H8A	0.9300
O1—H1A	0.8500	C10—C11	1.499 (3)
C1—C2	1.377 (4)	C10—H10A	0.9700
C1—C6	1.392 (3)	C10—H10B	0.9700
C1—H1B	0.9300	C11—C12	1.516 (3)
O2—C4	1.371 (3)	C11—H11A	0.9700
O2—H2A	0.8200	C11—H11B	0.9700
C2—C3	1.382 (3)	C12—C13	1.507 (3)
C2—H2B	0.9300	C12—H12A	0.9700
C3—C4	1.389 (3)	C12—H12B	0.9700
O3—C9	1.207 (3)	C13—C14	1.516 (4)
O4—C9	1.326 (3)	C13—H13A	0.9700
O4—C10	1.449 (3)	C13—H13B	0.9700
C4—C5	1.375 (3)	C14—C15	1.514 (4)
C5—C6	1.392 (3)	C14—H14A	0.9700
C5—H5A	0.9300	C14—H14B	0.9700
C6—C7	1.459 (3)	C15—H15A	0.9600
C7—C8	1.317 (3)	C15—H15B	0.9600
C7—H7A	0.9300	C15—H15C	0.9600
C8—C9	1.455 (3)		
			
C3—O1—H1A	118.8	O4—C10—H10B	110.4
C2—C1—C6	120.5 (2)	C11—C10—H10B	110.4
C2—C1—H1B	119.8	H10A—C10—H10B	108.6
C6—C1—H1B	119.8	C10—C11—C12	112.0 (2)
C4—O2—H2A	109.5	C10—C11—H11A	109.2
C1—C2—C3	120.9 (2)	C12—C11—H11A	109.2
C1—C2—H2B	119.6	C10—C11—H11B	109.2
C3—C2—H2B	119.6	C12—C11—H11B	109.2
O1—C3—C2	118.3 (2)	H11A—C11—H11B	107.9
O1—C3—C4	122.3 (2)	C13—C12—C11	113.9 (2)
C2—C3—C4	119.3 (2)	C13—C12—H12A	108.8
C9—O4—C10	117.5 (2)	C11—C12—H12A	108.8
O2—C4—C5	123.6 (2)	C13—C12—H12B	108.8
O2—C4—C3	116.7 (2)	C11—C12—H12B	108.8
C5—C4—C3	119.7 (2)	H12A—C12—H12B	107.7
C4—C5—C6	121.6 (2)	C12—C13—C14	114.1 (3)
C4—C5—H5A	119.2	C12—C13—H13A	108.7
C6—C5—H5A	119.2	C14—C13—H13A	108.7
C5—C6—C1	118.1 (2)	C12—C13—H13B	108.7
C5—C6—C7	119.2 (2)	C14—C13—H13B	108.7
C1—C6—C7	122.7 (2)	H13A—C13—H13B	107.6
C8—C7—C6	127.7 (2)	C15—C14—C13	113.5 (3)
C8—C7—H7A	116.1	C15—C14—H14A	108.9
C6—C7—H7A	116.1	C13—C14—H14A	108.9
C7—C8—C9	122.9 (2)	C15—C14—H14B	108.9
C7—C8—H8A	118.5	C13—C14—H14B	108.9
C9—C8—H8A	118.5	H14A—C14—H14B	107.7
O3—C9—O4	122.9 (2)	C14—C15—H15A	109.5
O3—C9—C8	125.4 (2)	C14—C15—H15B	109.5
O4—C9—C8	111.7 (2)	H15A—C15—H15B	109.5
O4—C10—C11	106.6 (2)	C14—C15—H15C	109.5
O4—C10—H10A	110.4	H15A—C15—H15C	109.5
C11—C10—H10A	110.4	H15B—C15—H15C	109.5
			
C6—C1—C2—C3	0.1 (4)	C5—C6—C7—C8	177.1 (3)
C1—C2—C3—O1	−179.5 (3)	C1—C6—C7—C8	−2.1 (5)
C1—C2—C3—C4	−0.7 (4)	C6—C7—C8—C9	−178.6 (3)
O1—C3—C4—O2	−0.1 (4)	C10—O4—C9—O3	−0.3 (4)
C2—C3—C4—O2	−178.9 (2)	C10—O4—C9—C8	179.3 (2)
O1—C3—C4—C5	179.9 (3)	C7—C8—C9—O3	0.9 (5)
C2—C3—C4—C5	1.1 (4)	C7—C8—C9—O4	−178.8 (3)
O2—C4—C5—C6	179.0 (2)	C9—O4—C10—C11	−175.1 (2)
C3—C4—C5—C6	−0.9 (4)	O4—C10—C11—C12	177.5 (2)
C4—C5—C6—C1	0.4 (4)	C10—C11—C12—C13	−177.4 (2)
C4—C5—C6—C7	−178.9 (2)	C11—C12—C13—C14	179.0 (3)
C2—C1—C6—C5	0.0 (4)	C12—C13—C14—C15	−175.2 (3)
C2—C1—C6—C7	179.3 (3)		

### Hydrogen-bond geometry (Å, °)

**Table:**

*D*—H···*A*	*D*—H	H···*A*	*D*···*A*	*D*—H···*A*
O1—H1A···O2	0.85	2.41	2.733 (2)	103
O1—H1A···O2^i^	0.85	2.07	2.857 (2)	154
O2—H2A···O3^ii^	0.82	1.97	2.786 (3)	173
C5—H5A···O3^ii^	0.93	2.54	3.243 (3)	133
C7—H7A···O3	0.93	2.56	2.874 (3)	100

Symmetry codes:  (i) −*x*+1, −*y*, −*z*+1; (ii) −*x*, −*y*+1, −*z*+1.
